# In Vitro Biocompatibility Assessment of Bioengineered PLA-Hydrogel Core–Shell Scaffolds with Mesenchymal Stromal Cells for Bone Regeneration

**DOI:** 10.3390/jfb15080217

**Published:** 2024-07-31

**Authors:** Federica Re, Luciana Sartore, Chiara Pasini, Matteo Ferroni, Elisa Borsani, Stefano Pandini, Andrea Bianchetti, Camillo Almici, Lorena Giugno, Roberto Bresciani, Silvia Mutti, Federica Trenta, Simona Bernardi, Mirko Farina, Domenico Russo

**Affiliations:** 1 Unit of Blood Diseases and Cell Therapies, Department of Clinical and Experimental Sciences, University of Brescia, “ASST-Spedali Civili” Hospital of Brescia, 25123 Brescia, Italy; silvia.mutti@unibs.it (S.M.); federica.trenta@unibs.it (F.T.); simona.bernardi@unibs.it (S.B.); mirko.farina@unibs.it (M.F.); domenico.russo@unibs.it (D.R.); 2 Centro di Ricerca Emato-Oncologica AIL (CREA), ASST Spedali Civili, 25123 Brescia, Italy; 3 University Center of Research “STem cells, bioENgineering and regenerative MEDicine”—STENMED, University of Brescia, 25123 Brescia, Italy; luciana.sartore@unibs.it (L.S.); chiara.pasini1@unibs.it (C.P.); matteo.ferroni@unibs.it (M.F.); elisa.borsani@unibs.it (E.B.); stefano.pandini@unibs.it (S.P.); andrea.bianchetti@asst-spedalicivili.it (A.B.); camillo.almici@asst-spedalicivili.it (C.A.); 4 Materials Science and Technology Laboratory, Department of Mechanical and Industrial Engineering, University of Brescia, 25123 Brescia, Italy; 5 Department of Civil, Environmental, Architectural Engineering and Mathematics (DICATAM), University of Brescia, Via Valotti 9, 25123 Brescia, Italy; 6 National Research Council (CNR)—Institute for Microelectronics and Microsystems, Via Gobetti 101, 40129 Bologna, Italy; 7 Division of Anatomy and Physiopathology, Department of Clinical and Experimental Sciences, University of Brescia, 25123 Brescia, Italy; lorena.giugno@unibs.it; 8 Interdepartmental University Center of Research “Adaption and Regeneration of Tissues and Organs (ARTO)”, University of Brescia, 25123 Brescia, Italy; 9 Laboratory for Stem Cells Manipulation and Cryopreservation, Department of Transfusion Medicine, ASST Spedali Civili di Brescia, 25123 Brescia, Italy; 10 Department of Molecular and Translational Medicine, University of Brescia, 25123 Brescia, Italy; roberto.bresciani@unibs.it; 11 Highly Specialized Laboratory, ASST Spedali Civili di Brescia, 25123 Brescia, Italy; 12 National Center for Gene Therapy and Drugs based on RNA Technology—CN3, 35122 Padua, Italy

**Keywords:** scaffold design, PLA, human mesenchymal stromal cells, gelatin–chitosan hydrogel, human platelet lysate, bone regeneration, tissue engineering, 3D printing

## Abstract

Human mesenchymal stromal cells (hMSCs), whether used alone or together with three-dimensional scaffolds, are the best-studied postnatal stem cells in regenerative medicine. In this study, innovative composite scaffolds consisting of a core–shell architecture were seeded with bone-marrow-derived hMSCs (BM-hMSCs) and tested for their biocompatibility and remarkable capacity to promote and support bone regeneration and mineralization. The scaffolds were prepared by grafting three different amounts of gelatin–chitosan (CH) hydrogel into a 3D-printed polylactic acid (PLA) core (PLA-CH), and the mechanical and degradation properties were analyzed. The BM-hMSCs were cultured in the scaffolds with the presence of growth medium (GM) or osteogenic medium (OM) with differentiation stimuli in combination with fetal bovine serum (FBS) or human platelet lysate (hPL). The primary objective was to determine the viability, proliferation, morphology, and spreading capacity of BM-hMSCs within the scaffolds, thereby confirming their biocompatibility. Secondly, the BM-hMSCs were shown to differentiate into osteoblasts and to facilitate scaffold mineralization. This was evinced by a positive Von Kossa result, the modulation of differentiation markers (osteocalcin and osteopontin), an expression of a marker of extracellular matrix remodeling (bone morphogenetic protein-2), and collagen I. The results of the energy-dispersive X-ray analysis (EDS) clearly demonstrate the presence of calcium and phosphorus in the samples that were incubated in OM, in the presence of FBS and hPL, but not in GM. The chemical distribution maps of calcium and phosphorus indicate that these elements are co-localized in the same areas of the sections, demonstrating the formation of hydroxyapatite. In conclusion, our findings show that the combination of BM-hMSCs and PLA-CH, regardless of the amount of hydrogel content, in the presence of differentiation stimuli, can provide a construct with enhanced osteogenicity for clinically relevant bone regeneration.

## 1. Introduction

Bone defects often result from trauma, revision arthroplasty, or tumor resection. Current bone reconstruction procedures include autologous, allogeneic, and xenogeneic bone grafts [1]. Due to the challenges associated with the current bone grafts, regenerative medicine has been integrated into clinical practice [2]. Regenerative medicine offers a revolutionary approach to tissue and organ repair, harnessing the power of human mesenchymal stromal cells (hMSCs) and three-dimensional (3D) scaffolds [3,4].

hMSCs are currently the most promising cell population for clinical applications in bone disease. They are being extensively studied for their potential in tissue engineering and regenerative medicine [5]. hMSCs are present in a multitude of tissues, including bone marrow, adipose tissue, and synovium [6]. hMSCs are multipotent adult stem cells with the remarkable ability to self-renew and differentiate into a variety of cell types within the mesodermal and other embryonic lineages, including osteocytes [7], neurons, muscle cells, hepatocytes, and epithelial cells [8,9]. However, the regenerative potential of hMSCs is intricately tied to their tissue of origin [10]. The potential efficacy of cell therapy based on hMSCs alone or in combination with scaffolds has been demonstrated in several clinical trials, although their efficacy remains limited [4]. Most of the current clinical trials on bone regeneration therapy have used bone-marrow-derived hMSCs (BM-hMSCs) [4,11,12,13]. Moreover, BM-hMSCs exhibit superior abilities for bone and cartilage formation using standard differentiation methods [14]. hMSCs stand out as the perfect cell source for various regenerative medicine applications with their unique properties: ease of isolation, ability to expand, differentiation capacity, self-renewal capacity, immunological properties, antimicrobial ability, and ability to migrate to injured sites [7,15,16,17,18]. In addition to these properties, autocrine or paracrine functions that generate growth factors have been proposed as the main mechanism that contributes to tissue repair [19,20].

Human platelet lysate (hPL) serves as an excellent substitute for fetal bovine serum (FBS) due to its rich content of growth factors, making it ideal for expanding hMSCs. By eliminating the risks of potential immune reactions to animal antigens, hPL allows for large-scale cell expansion for clinical use while satisfying all ISCT criteria [21]. hPL has proven to be highly effective in applications such as regenerative medicine, tissue engineering, cell culture, and cell therapy. However, its production remains controversial due to the numerous variables involved in the manufacturing process [22]. hPL stimulates proliferation, cell growth, and differentiation towards the osteogenic lineage of hMSCs from different sources [22].

Numerous biomaterials have undergone preclinical testing in conjunction with hMSCs for the purpose of bone regeneration [23]. The overall positive results of the bone regenerative medicine approaches have been confirmed by several approved clinical trials [24]. In most cases, frameworks made of ceramic cement were used, which merely fill bone defects [25]. However, bone graft substitutes should simultaneously fulfill several requirements, including biocompatibility, biodegradability, porosity, osteogenicity, osteoconductivity, and osteoinductivity to support the regeneration of bone tissue at the defect site by degrading in place and allowing newly formed bone to take its position [24]. For this reason, hybrid combinations of several classes of materials with different properties have received considerable attention in the inducement of osteogenic and chondrogenic differentiation [22,26,27,28,29].

Several composite bio-scaffolds, based on both synthetic and natural polymers, are potential candidates for bone tissue regeneration [30,31]. Synthetic polymers (e.g., polyethylene glycol, polyglycolic acid, poly-L-lactic acid (PLA), poly(ε-caprolactone) (PCL), and polyurethanes) allow for easier control of mechanical and degradation behaviors but are not bioactive, while bioactive ceramics (e.g., hydroxyapatite, corals, sulfate, tricalcium phosphate, bioactive glass, and calcium silicate) and natural polymers (e.g., chitosan, fibrin, hyaluronic acid, and collagen) mimic the extracellular matrix (ECM) of the bone but generally are mechanically weaker [2,30,32]. Current development is especially moving towards the use of hydrogels that mimic ECM excellently thanks to adequate porosity, surface morphology, and bioactive properties [33,34]. Natural biopolymers, such as Chitosan being added to the network of hybrid hydrogels, have an active role in promoting cell growth and osteo-differentiation [30,31,32,33,34,35]. Chitosan enhances osteogenesis in MSCs and upregulates osteopontin and collagen I [36]. Our recent studies showed higher stress relaxation in gelatin and CH-based scaffolds, which allow better cell adhesion, spreading, viability, and osteochondral differentiation [22,35,37]. However, given the complex functions of ECM and the fact that they are mineralized tissues, the mechanical properties of hydrogels are generally unsuitable for load-bearing applications [38]. Looking for enhanced mechanical support and higher control of mechanical properties and degradation behavior, stiffer polyesters such as PLA and PCL, which are already commercially available in medical grades, have been explored [39,40]. Based on these premises, the combination of synthetic polymers, such as PLA or PCL, with natural polymers, such as hydrogels, could lead to improvements of both the mechanical properties and bioactivity of the scaffolds; furthermore, the type and proportion of the components could be tailored for the specific purposes of tissue regeneration [41].

In a previous study, the authors developed a solvent-free fabrication process to create composite scaffolds. These scaffolds consist of a porous PLA-PCL core and a bioactive gelatin–CH hydrogel shell. It has been demonstrated that these scaffolds support the proliferation and osteogenic differentiation of BM-hMSCs [42]. However, more detailed knowledge and precise control of their structure–property correlations are required for the optimization of core–shell scaffolds [43]. Based on these results, the authors have recently utilized advanced additive manufacturing techniques to develop bioresorbable scaffolds with a core–shell architecture. These scaffolds are constructed using 3D-printed PLA cores with diverse lattice structures and are coated with gelatin–CH hydrogel. This innovative approach aims to fulfill the varied requirements for applications in bone tissue engineering [44,45,46].

In this study, the novel PLA-CH core–shell scaffolds were synthetized and investigated in more detail for bone tissue regeneration. We analyzed three different types of core–shell scaffolds with different core lattice structures, corresponding to different amounts of incorporated gelatin–CH hydrogel. The physical–mechanical and degradation properties of the scaffolds were assessed. In order to assess their biocompatibility and ability to replicate the natural bone microenvironment, the scaffolds were seeded with BM-hMSCs in the presence of FBS or hPL with or without differentiation stimuli. We then thoroughly evaluated the cell viability, proliferation, osteogenic differentiation, and scaffold mineralization.

## 2. Materials and Methods

### 2.1. Materials

For the scaffold core, poly(L-lactic acid) (PLA) filament was employed (Raise3D PLA Premium by Raise3D Technologies, Inc., Irvine, CA, USA). For the scaffold shell, the following materials were procured as the initial supply: poly(ethylene glycol) diglycidyl ether (PEGDGE) (molecular weight 526 Da) and chitosan (molecular weight 50,000 ÷ 190,000 Da, degree of deacetylation 75–85%) by Sigma-Aldrich Co (Milan, Italy); ethylene diamine (EDA) and acetic acid by Fluka (Milan, Italy). Pharmaceutical-grade type A gelatin (280 bloom, viscosity 4.30 mPs) was kindly supplied by ©Italgel S.p.A. (Cuneo, Italy). Dulbecco’s modified Eagle’s medium (DMEM), penicillin–streptomycin, l-glutamine, and sodium pyruvate were provided by Sigma-Aldrich Co (Milan, Italy). Amphotericin B and minimum essential medium (MEM) non-essential amino acid solution were purchased by Gibco, ThermoFisher Scientific (Milan, Italy).

### 2.2. Scaffold Fabrication

**Figure 1:**
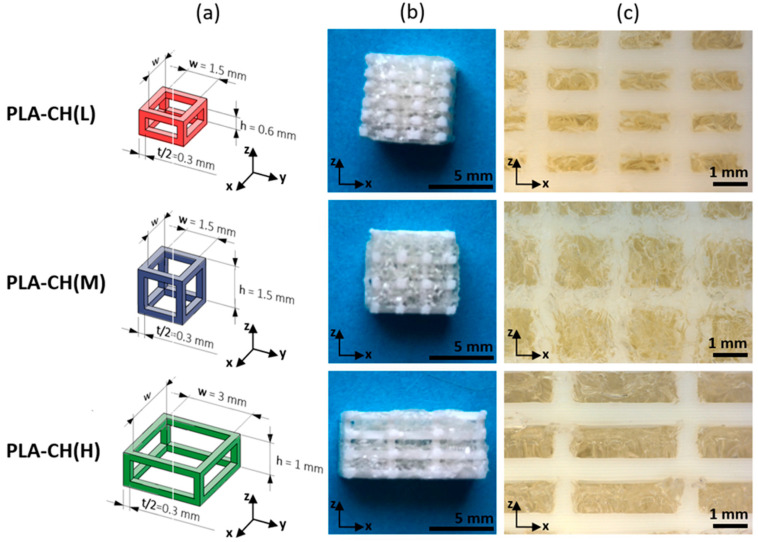
Geometry of lattice unit cells (**a**), dry specimens employed in in vitro experiments (**b**), and magnified details of wet specimens (**c**), relative to scaffolds hosting low (L), medium (M), or high (H) hydrogel content.

Lattice core structures with parallelepipedal unit cells were realized with the same strut thickness (*t* = 0.6 mm) but different hole height (h) and hole width (w), as indicated in Figure 1a. They were designed by using the software Solidworks (Dassault Systèmes, Vélizy-Villacoublay, France) and named after the amount of hydrogel they could contain (L: low; M: medium; H: high, corresponding to PLA-CH(L), PLA-CH(M), PLA-CH(H)). Cubic specimens with a side of about 10 mm, bar specimens with a cross-section of around 45–60 mm^2^, and a length of about 100 mm were 3D-printed by fused deposition modeling (FDM) of PLA. The slicing software Ideamaker and the 3D-printer Raise 3D Pro2 were both supplied by Raise3D Technologies, Inc. (Irvine, CA, USA). The following setup was employed: bed temperature of 60 °C; nozzle temperature of 205 °C; nozzle diameter of 0.2 mm; layer thickness of 0.1 mm (along the build direction z illustrated in Figure 1a).

The hydrogel shell was prepared by dissolving 6 g of gelatin in 65 mL of distilled water and successively adding 1.4 g of PEGDGE, 32.5 g of chitosan solution (2 wt% in acetic acid 1%), and 70 mg of EDA, resulting in a nominal composition of the final dry hydrogel equal to 74.3 wt% gelatin, 17.6 wt% PEGDGE, and 8.1 wt% chitosan. The process was carried out at 45 °C under mild magnetic stirring until the dissolution of the reactants and the initiation of crosslinking reactions between the epoxy groups of PEGDGE and the amino groups of gelatin and chitosan. The core structures were incorporated into the hydrogel forming solution at a temperature of 45 °C, and any entrapped air bubbles were removed through the application of three cycles of vacuum/air. After waiting for proper hydrogel crosslinking, the specimens were frozen and freeze-dried in a lyophilizer (HyperCOOL HC3055, LabTech Srl, Sorisole, Italy). The dry scaffolds were subjected to the removal of the excess hydrogel, post-curing in oven (2 h at 45 °C under vacuum), washing with distilled water, and a further freeze-drying treatment. In order to conduct the in vitro experiments, the bar specimens were cut along their length into slices of approximately 5 mm in thickness (Figure 1b), which were sealed in polypropylene bags under vacuum and sterilized by gamma irradiation (25 kGy of Cobalt 60 gamma rays, concordant to UNI EN ISO 1113 standard).

### 2.3. Characterization of Scaffolds

Physical–mechanical characterization and degradation experiments were carried out on cubic specimens. The void volume fraction (V_v_) of the core was determined through the equation below:

(1) $$V_{v}\left[\%\right]=\left(1-\frac{V_{c}}{V}\right)\times 100$$

where V_c_ = m_c_/ρ_c_ is the volume occupied by the lattice struts, m_c_ is the core mass (evaluated by weight), ρ_c_ is the PLA declared density [1], and V is the core total volume.

The hydrogel content by weight (Hy) and the water uptake (W) of the core–shell scaffolds were calculated as:

(2) $$Hy\left[\%\right]=\frac{m_{cs,dry}-m_{c}}{m_{cs,dry}}\times 100$$

(3) $$W\left[\%\right]=\frac{m_{cs,wet}-m_{cs,dry}}{m_{cs,dry}}\times 100$$

where m_cs,dry_ and m_cs,wet_ correspond to the mass of the composite specimens in dry conditions and after 24-h immersion in water, respectively. Core and core–shell specimens were subjected to mechanical tests at room temperature. The core–shell specimens have been previously immersed in distilled water at 37 °C for 24 h and for 7 weeks. A compressive load was applied perpendicularly to the z direction through an electromechanical dynamometer (Instron 3366 with 10 kN load cell, Illinois Tool Works Inc., Norwood, MA, USA), moving the crosshead at 2 mm/min. Compressive stiffness was evaluated as the apparent modulus (E_app_), derived from the initial slope of the stress–strain curves. The term apparent highlights that stress and strain are not those locally experienced by the material, but macromechanical parameters describing the response of the whole structure as an equivalent homogeneous material. Furthermore, the mass loss of the specimens was quantified following their immersion in distilled water at 37 °C for 28 days and subsequent drying under vacuum in an oven at 40 °C. The data were expressed as a percentage with respect to the initial mass of the dry scaffold, as well as with respect to the initial mass of the dry hydrogel shell only:

(4) $$mass\;loss/total\;mass\left[\%\right]=\frac{m_{cs,dry}-m_{deg}}{m_{cs,dry}}\times 100$$

(5) $$mass\;loss/hydrogel\;mass\left[\%\right]=\frac{m_{cs,dry}-m_{deg}}{m_{cs,dry}-m_{c}}\times 100$$

where m_deg_ is the mass of the scaffolds after hydrolytic degradation and drying.

### 2.4. Bone-Marrow-Derived Human Mesenchymal Stromal Cell (BM-hMSC) Culture

For the study, commercial human mesenchymal stromal cells derived from bone-marrow (BM-hMSCs) (PromoCell, Heidelberg, Germany) were expanded as previously reported [22] using a growth medium (GM) consisting of Dulbecco’s modified Eagle’s medium (DMEM), with glucose and 2% L-glutamine/penicillin–streptomycin/amphotericin B solution, MEM non-essential amino acids solution 1X, and 1 mM sodium pyruvate. GM was obtained by adding 10% of fetal bovine serum (GM FBS) or 5% of human platelet lysate (GM hPL). The cells were cultured in an incubator at 37 °C/5% CO_2_. hPL for the expansion of hMSCs was prepared according to standardized clinical procedures in closed systems as previously described [22]. Details of the technical procedure can be found in F. Re et al. [22].

### 2.5. BM-hMSC Seeding on Scaffolds

Once the BM-hMSCs reached 80% confluence, they were detached from the flask with trypsin ethylenediaminetetraacetic acid and neutralized with GM FBS. They were then centrifuged at 1100 rpm for 5 min and resuspended in GM before being counted. All experiments were performed with cells at passages 3 and 4. The dry scaffolds were used for cell seeding and placed in 24-well non-adherent plates (Corning, Sigma-Aldrich, St. Louis, MO, USA). The required cell number was concentrated in a volume of 50 μL. A small droplet (7 × 10^5^/50 μL viable BM-hMSCs) was applied to the top of the dry scaffold and left for 2 h in an incubator for complete absorption of the droplet. We have applied a static seeding method, namely “dry”, to increase the efficiency of cell penetration and distribution within the scaffold. Then, 1ml of GM FBS or GM hPL was added to each well. The medium for culturing the cells was changed three times per week. We examined each cell-seeded scaffold for cell viability and cell proliferation on day 28. On the day of the viability and proliferation analysis (day 28), the 24-well culture plates were replaced with new, sterile plates to remove any debris from the wells.

### 2.6. BM-hMSC Osteogenic Differentiation on Scaffolds

A cell suspension of 7 × 10^5^ viable BM-hMSCs was seeded onto the scaffold for osteogenic differentiation under static conditions. After 48 h, one milliliter of GM FBS or GM hPL was replaced with osteogenic medium (OM). For details, please referred to F. Re et al. [22]. Samples were also maintained in GM FBS or GM hPL without additional osteogenic factors for 4 weeks as controls. After 28 days of culture, the scaffolds were fixed with formaldehyde 4% for 1 h at 4 °C and dehydrated for subsequent analysis.

### 2.7. BM-hMSC Cell Viability and Cell Proliferation Assay

To assess the viability of BM-hMSCs in the scaffolds, a live/dead mammalian cell kit (ThermoFisher, Waltham, MA, USA) was applied following the manufacturer’s instructions. NucBlue^®^ Live Reagent (2 drops/mL) was added to the cultures for nuclei staining. Cells were analyzed using a Zeiss Observer Z1 fluorescence microscope. Several images of three different replicates of each sample were taken. Cell proliferation was determined using a Cell Counting Kit-8 (CCK-8, Sigma-Aldrich, St. Louis, MO, USA) on day 28 of cell culture according to the manufacturer’s instructions. The cell-cultured samples (three replicates) were incubated with the CCK-8 reagent at 37 °C for 2 h and 30 min. The absorbance of the supernatant that was transferred to a new cell culture plate was then measured at 450 nm using an Infinite 200 PRO plate reader (Tecan, Männedorf, Switzerland). The absorbance at 450 nm directly correlates with the number of viable cells in each sample.

### 2.8. Histological and Immunohistochemical Analysis

An automatic processor (Donatello series 2, Diapath S.p.A., Bergamo, Italy) was employed for the embedding of scaffolds in paraffin. A semi-automatic microtome, the Galileo semi-series 2 (Diapath S.p.A.), was employed to cut serial paraffin sections (7 µm thick) of each sample. The alternate sections were deparaffinized and rehydrated in accordance with standard procedures. Sections were then stained with a hematoxylin–eosin stain (automatic stainer Giotto; Diapath S.p.A.) for general morphological analysis. Moreover, the sections were evaluated histochemically, with von Kossa staining (Bio-Optica, Milan, Italy) for calcium deposits (brown/black dots) on PLA-CH sections, and immunohistochemically with a focus on the PLA-CH(M) sections for osteopontin (OSP), an early marker of osteoinduction, osteocalcin (OSC), a late marker of osteoinduction, bone morphogenetic protein-2 (BMP2), a marker of extracellular matrix remodeling, and collagen I. For immunohistochemistry, three alternate sections were incubated in primary antiserum anti-OSP (rabbit polyclonal antibody, 1:200, Abcam, Cambridge, UK), anti-OSC (mouse monoclonal antibody, 1:200, Santa Cruz Biotechnology, Dallas, TX, USA), anti-BMP-2 (goat polyclonal antibody, 1:200, Santa Cruz Biotechnology), and anti-collagen I (goat polyclonal antibody, 1:100, Santa Cruz Biotechnology). The sections were incubated in the primary antiserum, then in the appropriated biotinylated secondary antibodies, and, finally, in the avidin–biotin peroxidase complex (Vector Laboratories, Burlingame, CA, USA). The visualization of the immunopositivity reaction was achieved using a hydrogen peroxide and diaminobenzidine (chromogen) mixture, resulting in a brown coloration (Sigma, St. Louis, MO, USA). To ensure the accuracy of the results, an immuno-histochemical control was conducted by omitting the primary antibody and incubating the sections with non-immune rabbit serum and isotype-matched irrelevant rat IgGs, which served as a negative control. For all treatments, the staining for each antibody was performed simultaneously. Sections were counterstained with Carazzi’s hematoxylin (blue/violet color) to better visualize the positive reaction. The sections were dehydrated and mounted with DPX. The micrographs were performed using an optical microscope (Olympus BX50 Microscope, Hamburg, Germany) equipped with an image analyzer (Image-Pro Premier 9.3; 2018, Media Cybernetics, Rockville, MD, USA). For the analysis, the histochemical reaction was evaluated quantitatively, while the immunohistochemical reaction was evaluated qualitatively (positive vs. negative), both at a final 400× magnification, and digitally fixed images were analyzed blind. The analysis for each staining was performed on three sections in the center of each sample, spacing 50 mm each other, and considering three areas. The percentage of positive area within the scaffold meshes was evaluated for calcium deposits according to F. Re et al. (2021) [2]. As a control for histological staining, the scaffolds without cells were also analyzed.

### 2.9. Morphological and Microstructural Analysis

Scanning electron microscopy (SEM) was used to detect both the adhesion on all scaffold typologies and the differentiation toward the osteogenic lineage of the cells focusing on PLA-CH(M) sections. The samples were dehydrated through immersion in increasing alcohol solutions without any additional preparation. The scanning electron microscope (SEM) (ZEISS EVO LS-10, Oberkochen, Germany) was operated in environmental mode, with a pressure of 0.1–0.2 Torr at the specimen. The electron beam was accelerated to a range of 10–20 kV, and backscattered electron imaging was employed. In combination with SEM imaging, the elemental identification and localization provided by energy dispersive X-ray spectrometry (EDS) was used to pursue the identification of biomineral deposits. A Bruker SSD spectrometer was used for this purpose (Bruker, Billerica, MA, USA, Quantax 200 with a detector active area of 30 mm^2^). As a control for SEM analysis, the scaffolds without cells were also analyzed.

### 2.10. Statistical Analysis

The statistical analyses were conducted using two one-way analysis of variance (ANOVA) with Tukey’s multiple comparisons test. The numerical results are presented as mean ± standard deviation (SD). The graphical results were produced using GraphPad Prism (version 10). Three replicates of each sample were employed. The level of statistical significance was set at *p* < 0.05.

## 3. Results

### 3.1. Physical–Mechanical and Degradation Properties of the Scaffolds

The three types of core–shell scaffolds investigated are illustrated in Figure 1, showing their lattice unit cells (a) and their appearance in dry (b) and wet (c) conditions. In all the cases, the hydrogel is proved to be highly porous and to penetrate throughout the PLA lattice holes until the complete occupation of the core void volume. The lattices differ for the values of the width and height of their holes, corresponding to the different fractions of void volume and, therefore, to the different weight fractions of hydrogel in the final scaffolds, as reported in Table 1 for cubic specimens. The table also displays the overall water uptake of the specimens, being directly proportional to the hydrogel content since PLA water absorption can be neglected. The shell of the scaffolds is particularly important for the bioactive properties previously observed in the gelatin–chitosan hydrogels [37], of which it shares the same interconnected porous structure (~80% porosity) and water absorption (800% ca.).

Moreover, the three types of scaffolds present different mechanical and degradation properties (Table 2), which can be modulated by acting on the lattice design. In fact, as the core void volume fraction and the hydrogel content increase, the stiffness (apparent modulus) decreases, covering a range of values comparable with those of bone tissue [3]; the same results were found for both core and core–shell specimens, meaning that the hydrogel incorporation does not affect them. Furthermore, the PLA lattices were found capable of providing long-term mechanical support in the first phases of bone tissue regeneration, since the scaffolds maintained their stiffness after immersion in water at 37 °C for prolonged times (7 weeks). Conversely, the scaffold mass loss increases with the hydrogel content, as the shell earlier leaves space for the growth of new bone tissue, undergoing gradual hydrolysis in the aqueous environment (e.g., after 28 days in water at 37 °C, about 20 wt% of the hydrogel is degraded).

### 3.2. Assessment of In Vitro Viability, Proliferation, and Adhesion Capacity of hMSCs Cultured in PLA-CH(L), PLA-CH(M), and PLA-CH(H) Scaffolds

Following 28 days in either GM FBS or GM hPL, the viability of cells in the scaffolds was assessed in three replicates using a live/dead assay. Live cells were easily identifiable as they appeared green, a result of the enzymatic conversion of “calcein AM” to calcein (excitation 494 nm, emission 517 nm). Dead cells, on the other hand, emitted red fluorescence (excitation 517 nm, emission 617 nm) due to the binding of ethidium homodimer-1 to the nucleic acids of cells with damaged membranes. Both bright-field and fluorescence images were captured of the same area.

**Figure 2:**
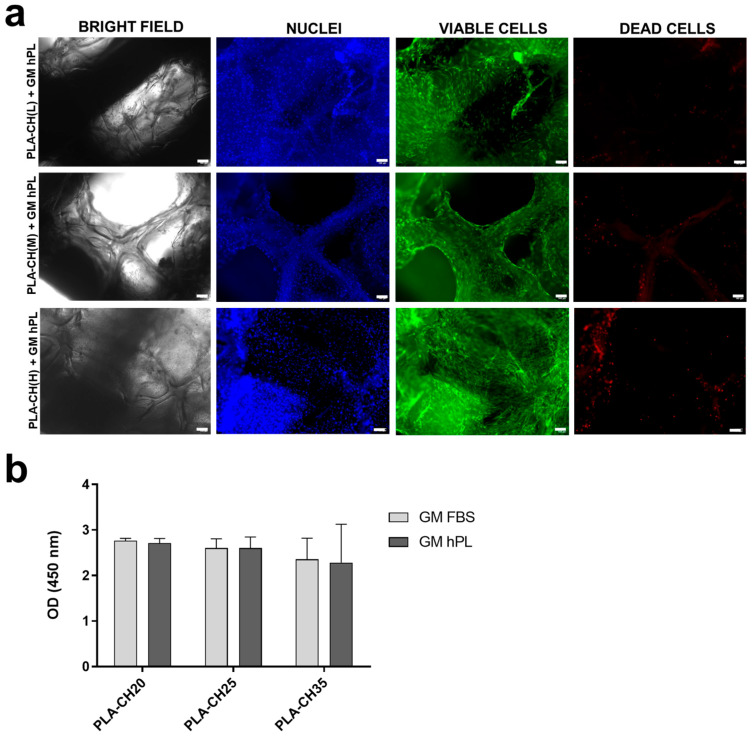
(**a**) Live/dead staining of BM-hMSCs cultivated in PLA-CH (L), PLA-CH(M), and PLA-CH(H) for 28 days in GM hPL. Scale bar: 100 μm. (**b**) Three-dimensional culture proliferation of BM-hMSCs cultivated in PLA-CH (L), PLA-CH (M), and PLA-CH (H) in the GM FBS or GM hPL at 28 days measured by the CCK8 assay.

The results from the fluorescence microscopy analysis of PLA-CH(L), PLA-CH(M), and PLA-CH(H) demonstrated the sustained viability of BM-hMSCs in the scaffolds after 28 days of culture, in both GM hPL (Figure 2a) and GM FBS (Supplementary Figure S1). The microscopic data clearly showed visible intact cell nuclei stained with DAPI (in blue), indicating a uniform distribution of cells within the scaffolds (in green), and a minimal presence of dead cells (in red) (Figure 2a, Supplementary Figure S1).

Cell proliferation was assessed by quantifying the number of viable cells within the scaffolds 28 days after cell seeding using the Cell Counting Kit-8 colorimetric assay (see Figure 2b). The CCK8 assay accurately detects viable cell activity. Previous studies have shown that CCK-8 solution permeates the entire scaffold without causing unspecific staining [47]. The testing showed no significant differences in cell proliferation with different scaffold types and culture media (GM FBS and GM hPL). In particular, comparisons between scaffolds with BM-hMSCs and GM FBS demonstrated no statistically significant differences after 28 days of culture (PLA-CH(L) vs. PLA-CH(M), *p*-value = 0.7013; PLA-CH(L) vs. PLA-CH(H), *p*-value = 0.4117; PLA-CH(M) vs. PLA-CH(H), *p*-value = 0.2487); the comparative analysis of scaffolds with BM-hMSCs and GM hPL did not reveal any statistically significant differences (PLA-CH(L) vs. PLA-CH(M), *p*-value = 0.1123, PLA-CH(L) vs. PLA-CH(H), *p*-value = 0.4307, PLA-CH(M) vs. PLA-CH(H), *p*-value = 0.3183); no significant differences were observed between the same scaffolds in GM FBS or GM hPL (PLA-CH(L) GM FBS vs. PLA-CH(L) GM hPL, *p*-value = 0.3481; PLA-CH(M) GM FBS vs. PLA-CH(M) GM hPL, *p*-value = 0.3481; PLA-CH(H) GM FBS vs. PLA-CH(H) GM hPL, *p*-value = 0.3481). The mean absorbance values, as determined by the CCK-8 assay after a 28-day incubation period, were 2766 ± 0.050 for PLA-CH(L) GM FBS; 2603 ± 0.203 for PLA-CH(M) GM FBS; 2354 ± 0.465 for PLA-CH(H) GM FBS; 2713 ± 0.100 for PLA-CH(L) GM hPL; 2600 ± 0.244 for PLA-CH(M) GM hPL; 2282 ± 0.844 for PLA-CH(H) GM hPL. Therefore, we considered the scaffolds to be equivalent irrespective of the cell proliferation in the different cultures.

**Figure 3:**
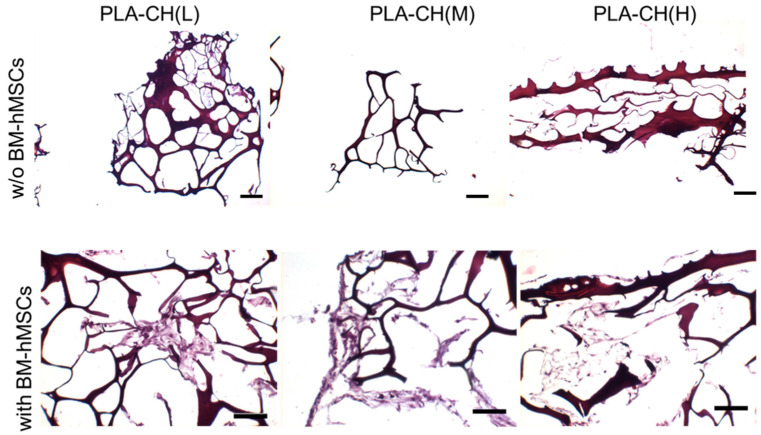
BM-hMSC viable cells cultivated for 28 days in scaffolds in GM hPL, examined by hematoxylin–eosin staining. Scale bar: 100 μm.

To better assess the scaffold morphology and cell colonization, H&E studies were accomplished on PLA-CH(L), PLA-CH(M), and PLA-CH(H) seeded with BM-hMSCs and grown in GM FBS and GM hPL. The scaffolds showed affinity for staining and revealed heterogeneous large pores. The cells appeared well integrated into the scaffold with an elongated morphology creating a 3D network in the pores and settled on the meshes of the hydrogel (Figure 3, Supplementary Figure S2) but not on the PLA surface without a preference for the scaffold type.

**Figure 4:**
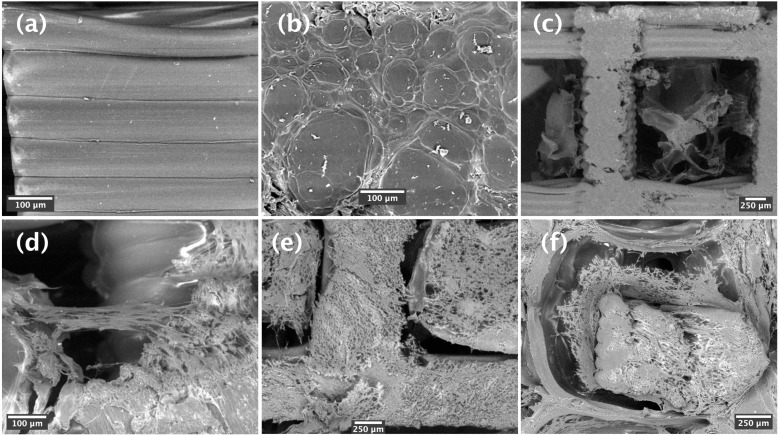
SEM images of PLA-CH(M) scaffolds. (inset (**a**)): the PLA structure; (inset (**b**)), detail of the hydrogel structure; (inset (**c**)), view of the entire structure. SEM images of cellular growth over the scaffolds with BM-hMSCs in GM hPL: PLA-CH(L), PLA-CH(M), and PLA-CH(H) in the inset (**d**–**f**) respectively.

In addition, the morphology, adhesion, and dispersion of BM-hMSCs on the PLA-CH(L), PLA-CH(M), and PLA-CH(H) samples were observed with SEM after 28 days of culture in vitro. In fact, the scaffold surface was essential for effective cell adhesion. Figure 4a–c shows the framework of the scaffolds, with detailed information on PLA, hydrogel, and the overall structure. Figure 4 and Supplementary Figure S3 show the interaction of the cells with the scaffolds and their adhesion and spreading preferentially in the pores of the hydrogel shell compared to the PLA core structure, where the cells were mainly localized on the rough surface of the sectioned PLA struts (Figure 4d–f). BM-hMSCs adhered to the hydrogel shell, even bridging the open porosity. The cells extended on a large area, were predominantly elongated, and had a fibroblast-like morphology with evidence of cytoplasmic processes facilitating adhesion and cell communication. These results clearly demonstrate that BM-hMSCs exhibit a remarkable affinity for attachment, proliferation, and migration in PLA-CH(L), PLA-CH(M), and PLA-CH(H) scaffolds, with no significant variations observed between the different scaffolds.

### 3.3. Analysis of the Osteogenic Differentiation and Mineralization of the Scaffolds Seeded with Osteo-Differentiated BM-hMSCs

#### 3.3.1. Histochemical and Immunohistochemical Stainings

**Figure 5:**
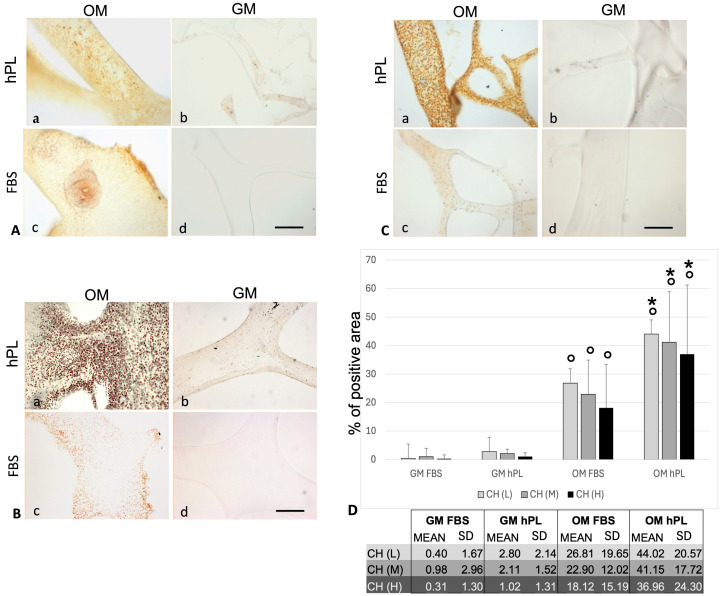
Calcium deposit distribution in hydrogels PLA-CH cultured with cells using von Kossa staining (brown/black dots). Microphotographs of scaffolds with differentiated (**a**,**c**) and undifferentiated (**b**,**d**) BM-hMSCs (400× magnification, scale bar: 40 µm) in (**A**) PLA-CH(L), (**B**) PLA-CH(M), and (**C**) PLA-CH(H). Calcium deposits appeared as dots in brown/black color. Quantification of percentage of positive area (**D**) within scaffold meshes; ° *p* < 0.001 vs. respective GM; * *p* < 0.001 vs. OM FBS. No statistical differences were observed when comparing the scaffolds with the same treatment.

**Figure 6:**
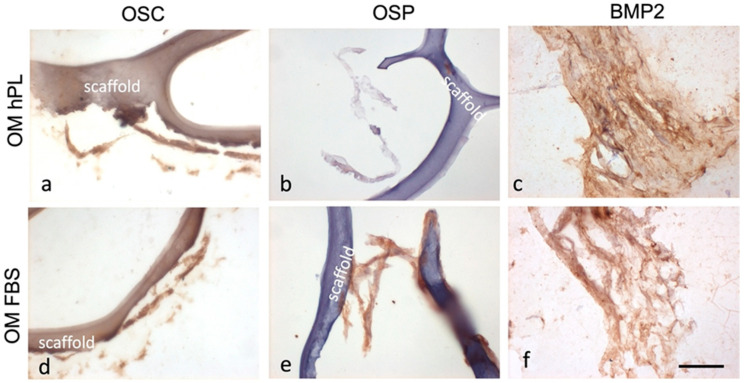
Micrographs of the hydrogels PLA-CH(M) with differentiated (OM) BM-hMSCs in hPL (**a**–**c**) and FBS (**d**–**f**) immunostained for OSC (**a**,**d**), OSP (**b**,**e**), and BMP2 (**c**,**f**) (brown color) with hematoxylin counterstaining (blue/violet) (400× magnification, bar 40 µm).

The results demonstrated the presence of calcium deposits (von Kossa stain) in the structures of the differentiated scaffolds (Figure 5). In particular, the calcium deposits showed a higher presence in the scaffold cultured with OM hPL with respect to GM (*p* < 0.0001) and OM hPL with respect to OM FBS (*p* < 0.0001 for CH(M) and CH(H); *p* = 0.0002 for CH(L)). In GM, a minimal presence of calcium deposits has been evidenced, especially in GM hPL. The three scaffolds did not show statistical significance among them related to the condition tested. Regarding the immunohistochemical evaluation focusing on scaffold PLA-CH(M), the cells appeared well integrated in the scaffold and produced an extracellular matrix with a positivity for collagen I (Supplementary Figure S4). Figure 6 shows a clear differentiation state as osteoblasts with a positivity for OSC in OM hPL. Moreover, the immunopositivity of the differentiated cells with hPL for OSC, but not for OSP, showed a consolidated differentiation state as osteoblasts in this treatment. On the contrary, the immunopositivity of the differentiated cells with FBS for OSP, which showed a delay in the differentiation process, confirmed a lower presence of calcium deposits. Finally, the immunopositivity for BMP2 in both differentiated groups indicated a remodeling activity of the extracellular matrix.

#### 3.3.2. SEM–EDS Analysis of Scaffold Mineralization

**Figure 7:**
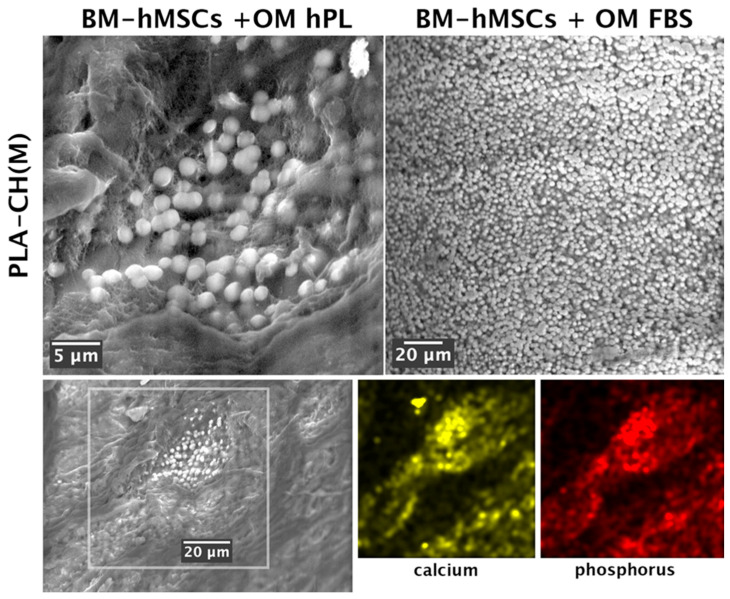
SEM images and EDS investigation of calcium phosphate deposition in PLA-CH(M) with BM-hMSCs in the OM hPL and OM FBS at day 28. Upper part: the bright round particles observed on both samples (upper part of the figure) are ascribed to hydroxyapatite formation. Lower part: evaluation of calcium and phosphorous with SEM–EDS of PLA-CH(M) with BM-hMSCs in OM with FBS at day 28. The mapping of Ca and P in the boxed area (78 × 78 µ^2^) indicates the extended presence of these elements below the biological film visible at the surface.

SEM investigation confirmed that the PLA-CH(M) scaffold supported cell differentiation towards the osteogenic lineage by calcium phosphate deposition (Figure 7). BM-hMSCs differentiated into osteoblast cells with evidence of preferential calcium phosphate deposition on the hydrogel shell compared to the PLA core. Calcium phosphate deposition was present using osteogenic induction media, both with hPL and FBS, in the PLA-CH(M) seeded with BM-hMSCs. Significant mineral deposits were visible within the scaffolds, particularly in the porous structure of the hydrogel shell, which was ideal.

To confirm the presence of mineral deposits in the PLA-CH scaffolds bioengineered with BM-hMSCs, SEM–EDS compositional analysis was performed at 28 days. This analysis conclusively demonstrates that the inorganic phase is predominantly composed of calcium and phosphorus, the key elements in hydroxyapatite microparticles formed during osteogenic differentiation. The samples incubated in an osteogenic medium showed the detection of both calcium and phosphorous. The chemical distribution maps collected clearly indicate that these elements co-localize in the same areas of the sections (Figure 7, lower part). In a previous publication [28], the mineral deposits have been deeply investigated through a correlative approach of SEM–EDS and optical microscopy coupled to Raman spectroscopy. It has been demonstrated that similar mineral deposits are indeed Ca-P-O compounds with hydroxyapatite features in the Raman spectra. This correlative technique features more significant spatial resolution than XRD or FTIR and could be successfully implemented for the detection of the early stage of the mineralization process. In the present work, the three-dimensional spatial arrangement of the scaffold and the significant fluorescence from the PLA affected the collection of the Raman signal and the consequent evidence of hydroxyapatite formation. Based on this previous analysis, the bright, round particles visible in Figure 7 could be confidently identified as hydroxyapatite mineral deposits, owing to the combination of SEM image contrast and the simultaneous detection of Ca and P from EDS. On the contrary, the large, irregular detail visible in the SEM image of Figure 7 and in the elemental mapping of Ca only was not considered as a hydroxyapatite.

The evidence of calcium and phosphorous ions was shown in the presence of both FBS and hPL as supplements in the osteogenic culture media. The scaffolds seeded with BM-hMSCs treated with GM FBS or GM hPL, as well as the unseeded scaffold, did not show any significant presence of calcium or phosphorous. This demonstrates that these chemical elements primarily accumulated in the scaffolds seeded with the cells that were grown in OM.

## 4. Discussion

Stem-cell-based therapy is a crucial topic in regenerative medicine, with hMSCs playing a significant role in combination with three-dimensional biocompatible scaffolds [48]. hMSCs have been demonstrated to be appropriate for the treatment of bone tissue in regenerative medicine [49]. At the same time, there is increasing interest in biocompatible materials that offer an adequate environment for cell growth and differentiation and are, therefore, fundamental in increasing the efficiency in healing injured bone and bone regeneration [27]. While numerous tissue engineering strategies have been developed and researched in detail, only a few approaches have translated into clinical applications [4].

In previous work, the authors have developed 3D gelatin–chitosan hybrid hydrogels in combination with hMSCs to improve osteogenic and chondrogenic differentiation [22,29]. However, the mechanical properties of hydrogels are much lower than those of bone and generally unsuitable for temporary load bearing. Looking for stiffer bioresorbable polymers commercially available in medical grades and offering better control over their mechanical and degradation behaviors, synthetic polymers, such as PCL, PLGA, and PLA, are often used for bone tissue engineering [38,50]. PLA, an FDA-approved biodegradable synthetic polymer, is widely utilized in various biomedical applications due to its biocompatible and biodegradable properties [51]. In bone tissue regeneration systems, PLA is subjected to diverse fabrication methods involving the modification of polymer structures, the development of blended polymer fibers, and the integration of nanoparticles to create nanocomposite fibers [52]. Particularly, given the bioactive role of hydrogels on one side and the mechanical support offered by PLA on the other, combining these two materials improves both the mechanical properties and the bioactivity of the scaffolds [41]. Hybrid combinations of materials have received considerable attention in the inducement of osteogenic and chondrogenic differentiation [52]. Recently, the authors have developed bioresorbable scaffolds with an innovative core–shell architecture. These scaffolds are based on 3D-printed PLA cores with different lattice structures and grafted with CH-hydrogel to meet the diverse requirements for bone tissue engineering applications [44].

The goal was to create a hybrid structure that imitates natural bone tissue, customizing the flexible multi-material design to achieve a good balance between mechanical support and regenerative potential. In order to provide the scaffolds with complementary functions, two components were carefully chosen. The PLA core was designed with lattice structures of varying void volume fractions, allowing for precise adjustment of stiffness and strength. The hydrogel filled the entire void volume of the core lattices with a highly porous shell, which presented highly interconnected pores, smaller than the holes of the core and offering a greater surface area for cell adhesion and homogeneous colonization. The PLA core aimed at transiently providing a tailored mechanical device for bone tissue, while the hydrogel shell served as a microenvironment for cell colonization and mineralization and underwent degradation, leaving space for the formation of new bone tissue [44]. An extensive mechanical characterization of scaffolds with a variety of lattice structures highlighted that their compressive stiffness and strength can be modulated over a wide range of values by acting on the lattice geometrical parameters, aiming at matching the properties of specific target tissues [53].

Here, three distinct PLA-hydrogel core–shell scaffolds with varying hydrogel contents were created and thoroughly examined to determine the conditions for bone tissue regeneration in vitro. The scaffolds had a core–shell structure. The lattice core was realized by additive manufacturing, while the shell was enclosed throughout the core by inserting and crosslinking a hydrogel-forming solution. The hydrogel network created an open porous structure with heterogeneous pore size (around 100 µm on average) that covered and stuck to the struts of the PLA lattice (Figure 4). By changing the size of the lattice unit cells, core void volume fractions between about 69% and 83% were obtained, allowing us to host hydrogel contents between about 12 wt%, for PLA-CH(L), and 22 wt%, for PLA-CH(H) (Table 1). While the hydrogel content increased with the core void volume fraction, the mechanical properties were higher for denser lattices (Table 2). More in detail, the highest stiffness (about 380 MPa) was obtained for the lattices with the smallest holes and lowest void volume fraction (69% ca.), whereas the lowest modulus (about 180 MPa) was achieved for those with the largest holes and highest void volume fraction (83% ca.). In other words, the PLA structure density and the lattice design determined the stiffness values of the three scaffolds. Furthermore, PLA-CH exhibited a compressive stiffness that was significantly higher than that of the hydrogel alone, remaining unaffected by the hydrogel incorporation, and surpassing several composite or core–shell bioresorbable scaffolds that had been suggested in the literature for the objective of bone tissue engineering (Table 3). Therefore, the PLA-CH core–shell scaffolds are a very promising bioresorbable tool for reconstructing hard tissues. While PLA maintained the stiffness stable for several weeks in water at body temperature, the hydrogel gradually lost mass due to hydrolytic degradation, leaving space for new bone tissue formation (Table 2).

To analyze the capability of the core–shell scaffolds to simulate the natural microenvironment of the bone, the scaffolds were seeded with BM-hMSCs in different culture conditions. According to previous works, cells were grown in a culture medium containing FBS or hPL as a supplement [22,28]. To induce the osteogenic differentiation, additional stimuli were added as previously described [22]. The analyses were performed after 4 weeks of culture, both in growth medium and differentiation medium, as generally occurred [61].

The viability assay confirmed that all the cells were proliferating and viable as required after 28 days of culture (Figure 2). The cells were anchored to the area of the scaffold, preferentially in the hydrogel pores rather than the PLA structure (Figure 3 and Figure 4). The homogeneous cell distribution of the hydrogel was better appreciated with SEM analysis (Figure 4), compared to the histological approach (Figure 3), probably due to the technical procedure that could lead to the loss of some cells adhered to the structure of scaffolds. However, histological staining allows us to better reveal the capacity of BM-hMSCs to cluster in certain regions of the scaffold, preferentially in the lower size pores and on the surface of the scaffolds, homogeneously covering them preferentially in the outer region compared to the inner region of the scaffolds (Figure 3). No significant difference in cell proliferation was observed in the three types of core–shell scaffolds and different culture media (GM FBS and GM hPL), even if a trend favoring a decrease could be observed when PLA-CH(H) was seeded with BM-hMSCs and analyzed after 28 days of culture (Figure 2). This is probably due to a slight dilution of the test due to the increased hydrogel content in the void volume fraction. Calcium deposition was detected in BM-hMSCs seeded in GM hPL, though having a very low concentration, while GM FBS was inefficient (Figure 2). It is noteworthy that a cell culture procedure using hPL, instead of FBS, stimulated both cell expansion and differentiation [62,63]. Previous works emphasize the role of hPL in increasing the osteogenic and chondrogenic differentiation capabilities of BM-hMSCs without requiring additional differentiation stimuli in hydrogels [22,28,29].

To induce the differentiation of BM-hMSCs toward osteogenic lineage in the core–shell scaffolds, additional differentiation stimuli were added to the culture medium. The calcium deposits were primarily observed using a histochemical method (von Kossa staining), and, subsequently, protein markers for osteogenic differentiation were highlighted using an immunohistochemical technique, both for optical microscopy evaluation. In detail, OPN is an early and pivotal osteogenic marker for the expression of secondary late markers [64], such as OSC [65,66,67]. The greater presence of deposits was associated with the consolidated, differentiated state of osteoblast (Figure 5), which was confirmed by the OSC immunopositivity and OSP immunonegativity of OM hPL (Figure 6). In addition, the delay in the differentiation state of cells cultured in OM FBS was associated with the presence of immunopositive cells for OSC and OSP. In both cases, BMP-2 was activated for extracellular matrix remodeling (production of collagen I), and bone regeneration itself confirmed its essential role during this process [68]. Finally, these data confirmed the high potentiality of hPL in a regenerative context. The chemical elements analyzed with SEM–EDS confirmed the accumulation of granular deposits only in the scaffolds seeded with cell growth in OM according to the von Kossa staining (Figure 7). No mineral deposits were found when BM-hMSCs grew in the scaffold in basal conditions, without adding differentiation stimuli (Figure 4). After 28 days of osteogenic induction, the formation of granular deposits was found in the core–shell scaffolds, preferentially on the hydrogel surface and between its porosities, while no mineralization was observed on the PLA lattice structure (Figure 7). EDS spectra associated with SEM revealed that the mineral deposits were composed of calcium and phosphorous ions, and these elements co-localized in the same areas of the sections, confirming the presence of hydroxyapatite. The co-localization of calcium and phosphorus suggests an outstanding ability of the core–shell scaffolds to imitate the physiological environment, as already previously demonstrated in gelatin–chitosan hydrogels [22].

## 5. Conclusions

In this study, bioresorbable scaffolds with innovational core–shell architecture were prepared by grafting a gelatin–chitosan (CH) shell in 3D-printed PLA cores with different lattice structures and investigated in more detail for their biocompatibility and ability to induce bone tissue regeneration in vitro. The objective was to develop a hybrid structure that mimics native bone tissue, creating a versatile multi-material design that is essential for achieving an optimal balance between mechanical support and regenerative potential. Two components were selected to provide the scaffolds with complementary functions: a PLA core, designed with lattice structures and different void volume fractions, and a hydrogel shell that filled in the entire void volume of the core lattices. The PLA core aims to supply tailored mechanical support for bone tissue in the long term, and the hydrogel shell exhibits the favored microenvironment for cell expansion. Overall, our results demonstrated that PLA-CH(L), PLA-CH(M), and PLA-CH(H), differing in the amount of hydrogel content, are biocompatible, and their morphological, mechanical, and degradation properties are appropriate to support cell adhesion, growth, proliferation, and osteogenic differentiation. Additionally, the amount of hydrogel content nor the geometry of the core–shell structure showed influence on the cell behavior. For the cell culture procedure, the cell osteogenic differentiation results were obtained using both hPL and FBS with the addition of differentiation stimuli in the cell culturing. A consolidated differentiation state of BM-hMSCs, as osteoblasts, has been shown to perform better with hPL compared with FBS. Indeed, the cells cultured in differentiation medium with FBS also showed a calcium and phosphorous deposition but with a lower expression. The formation of granular deposits was observed in PLA-CH(M) seeded with osteo-differentiated BM-hMSCs, both in OM FBS and OM hPL. These results suggest that the combination of BM-hMSCs and PLA-CH in the presence of osteogenic differentiation stimuli can provide a construct for guiding bone tissue regeneration. In fact, the scaffolds analyzed presented different mechanical and degradation properties but similar biocompatibility, so they could be used in different clinical settings relating to the anatomical site required for bone regeneration.

## Figures and Tables

**Table 1:** Geometry and composition of cubic scaffolds hosting low (L), medium (M), or high (H) hydrogel content: width (w) and height (h) of lattice holes; void volume fraction in the core; hydrogel content; water uptake after 24 h in water.

Label	w × h [mm]	Core Void Volume Fraction [%]	Hydrogel Content [%]	Water Uptake (24 h) [%]
PLA-CH(L)	1.5 × 0.6	68.8 ± 0.3	11.6 ± 0.4	103 ± 7
PLA-CH(M)	1.5 × 1.5	77.0 ± 0.3	17.6 ± 0.4	137 ± 4
PLA-CH(H)	3.0 × 1.0	83.4 ± 0.2	22.0 ± 0.9	201 ± 9

**Table 2:** Mechanical and degradation properties of cubic scaffolds hosting low (L), medium (M), or high (H) hydrogel content: apparent modulus (E_app_) of core specimens, core–shell scaffolds in wet conditions (24 h immersion), and core–shell scaffolds maintained in water at 37 °C for 7 weeks; mass loss of core–shell scaffolds maintained in water at 37 °C for 28 days.

Label	E_app_, Core [MPa]	E_app_, Core-Shell [MPa]	E_app_, Core-Shell 7 Weeks [MPa]	Mass Loss, Core-Shell 28 d [%]
PLA-CH(L)	381 ± 14	378 ± 17	367 ± 39	11.4
PLA-CH(M)	223 ± 34	230 ± 6	231 ± 6	6.8
PLA-CH(H)	204 ± 1.3	174 ± 7	179 ± 6	5.4

**Table 3:** Compressive stiffness of various hydrogel-based scaffolds.

Material Components *	Void Volume of the Stiff Component [%]	Compressive Modulus [MPa]	Ref.
Gelatin-chitosan (CH) hydrogel	not applicable	0.04 ÷ 0.25	Dey K. et al. [37]
PLA core + CH-hydrogel shell	40 ÷ 90	50 ÷ 550	Pasini C. et al. [54]
Calcium-deficient hydroxyapatite + alginate hydrogel	not available	7	Raja N. et al. [55]
PLA + gelatin methacrylate hydrogel + gold nanoparticles	74 ÷ 87	300 ÷ 700	Heo D.N. et al. [43]
PLA/PVA + chitosan hydrogel + hydroxyapatite	not available (>80)	0.7 ÷ 1.2	Li T.T. et al. [56]
PCL + gelatin-heparin cryogel	80	0.004	Lee S.S. et al. [57]
PCL + nanohydroxyapatite + collagen	50	90 ÷ 110	Cho Y.S. et al. [58,59]
PCL + peptide-based hydrogel	45	11	Wu T. et al. [60]

* Note that particulate composites were excluded from the examples due to the lower mechanical properties typically offered by discontinuous reinforcement.

## Data Availability

The original contributions presented in the study are included in the article/Supplementary Material, further inquiries can be directed to the corresponding author/s.

## Supplementary Materials

The following supporting information can be downloaded at: https://www.mdpi.com/article/10.3390/jfb15080217/s1. Figure S1: Live/dead staining of BM-hMSCs cultivated in PLA-CH (L), PLA-CH(M), and PLA-CH(H) for 28 days in GM FBS. Scale bar: 100 μm. Figure S2: BM-hMSCs viable cells cultivated for 28 days in scaffolds in GM FBS examined by hematoxylin–eosin staining. Scale bar: 100 μm. Figure S3: SEM images of PLA-CH(L), PLA-CH(M), and PLA-CH(H) with BM-hMSCs in GM FBS. Figure S4: Micrographs of the hydrogels PLA-CH(M) with differentiated (OM) BM-hMSCs in hPL (a) and FBS (b) immunostained for collagen I (Coll I) (brown color) with hematoxylin counterstaining (blue/violet) (400× magnification, bar 40 µm).
