# Supplementary material for: In Vitro Biocompatibility Assessment of Bioengineered PLA-Hydrogel Core–Shell Scaffolds with Mesenchymal Stromal Cells for Bone Regeneration
Source: J Funct Biomater. 2024 Jul 31;15(8):217. doi: 10.3390/jfb15080217 (PMC11355418; doi:10.3390/jfb15080217)
Supplement: Supplementary file 1 [file jfb-15-00217-s001.zip › jfb-3105145-supplementary.pdf]

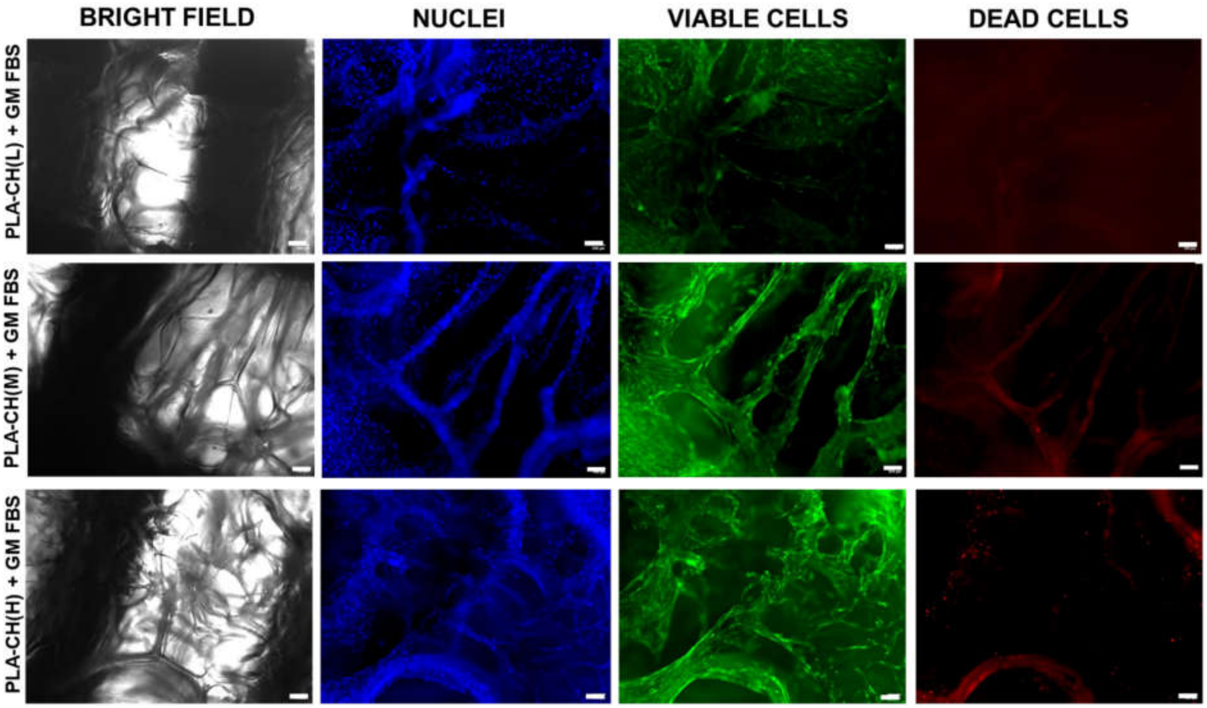

Supplementary Figure S1. Live/dead staining of BM-hMSCs cultivated in PLA-CH (L), PLA-CH(M), PLA-CH(H) for 28 days in GM FBS. Scale bar: 100 μm.

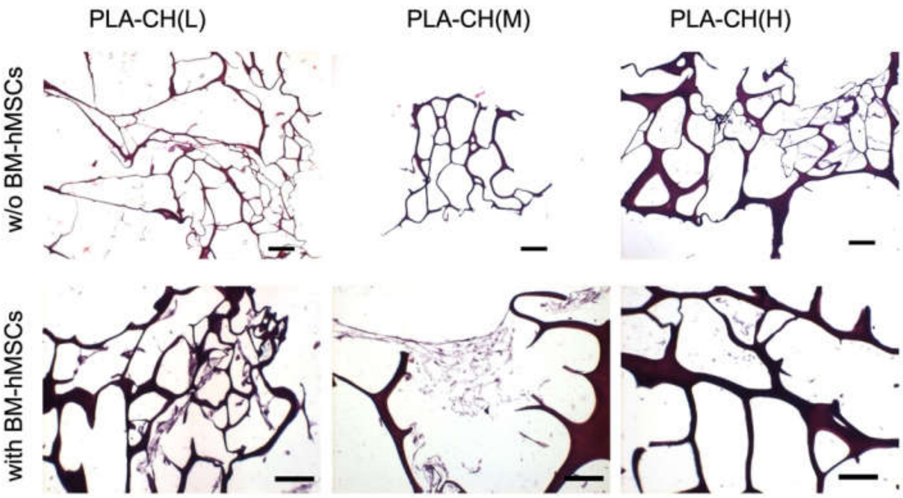

Supplementary Figure S2. BM-hMSCs viable cells cultivated for 28 days in scaffolds in GM FBS examined by hematoxylin-eosin staining. Scale bar: 100 μm.

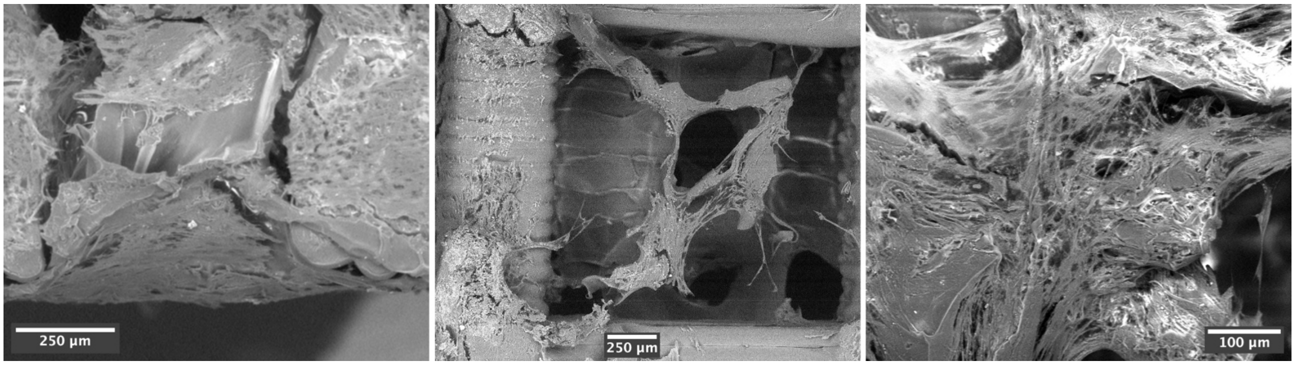

Supplementary Figure S3. SEM images of PLA-CH(L), PLA-CH(M), PLA-CH(H) with BM-hMSCs in GM FBS.

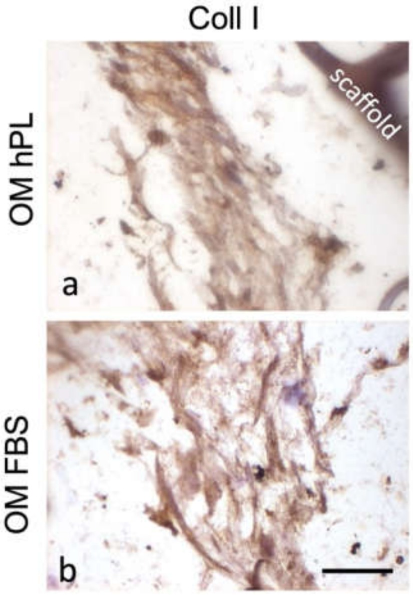

Supplementary Figure S4. Micrographs of the hydrogels PLA-CH(M) with differentiated (OM) BM-hMSCs in hPL (a) and FBS (b) immunostained for collagen I (Coll I) (brown color) with hematoxylin counterstaining (blue/violet) (400X magnification, bar 40 µm).
